# Energy, exergy, environment and economic (4E) analysis of PV/T module assisted vapor compression refrigeration system: An experimental study

**DOI:** 10.1016/j.heliyon.2024.e37690

**Published:** 2024-09-10

**Authors:** Gökhan Yıldız, Ali Etem Gürel, Ferzan Katırcıoğlu, Ümit Ağbulut

**Affiliations:** a Department of Electronics and Automation, Düzce Vocational School, Düzce University, 81010, Düzce, Türkiye; b Department of Electricity and Energy, Düzce Vocational School, Düzce University, 81010, Türkiye; c Department of Mechatronic Engineering, Faculty of Engineering, Düzce University, 81620, Düzce, Türkiye; d Department of Mechanical Engineering, Mechanical Engineering Faculty, Yildiz Technical University, Istanbul, 34349, Türkiye; e Department of Technical Sciences, Western Caspian University, Baku, Azerbaijan; f Department of Chemistry and Biochemistry, California State University, Los Angeles, State University Drive 5151, 90032, Los Angeles, CA, United States

**Keywords:** Thermodynamic analysis, Photovoltaic/thermal module, Vapor compression refrigeration system, Superheating

## Abstract

Despite the rise in the prices of fossil fuels, the increase in their demand, and damaging the environment, a large part of the world's energy needs have been today met by fossil fuels. In this direction, interest in renewable energy sources has increased. Solar energy stands out among renewable energy sources because it is endless and clean. However, today, the use of solar energy is not used alone, but in combination with other thermal energy systems. In building applications, it is mostly used in heating, refrigeration, and HVAC systems, which have a high part in energy consumption. In this study, the solar energy and cooling system were not used separately as in previous studies but were used as a hybrid. The focus was on increasing the performance of both systems by operating them together. In this study, energy, exergy, thermoeconomic, and environmental analyses were applied to the PV/T-assisted vapor compression refrigeration system (PV/T-VCRS) at different storage temperatures (25 °C, 30 °C, and 35 °C). As a result, an 8.5 % lower surface temperature of the module in PV/T-VCRS 25 °C was measured compared to PV/T-VCRS 30 °C and 35 °C. In direct proportion to the module surface temperatures, 13 % better electrical efficiency was obtained in PV/T-VCRS 25 °C compared to 30 °C and 35 °C. The COP value increased by 15.46 % in PV/T-VCRS 25 °C compared to 30 °C and 35 °C. A 13 % improvement in exergy efficiency was observed in PV/T-VCRS 25 °C compared to 30 °C and 35 °C. The enviroeconomic parameter PV/T-VCRS is calculated as 15.17 ¢/h, 16.52 ¢/h, and 17.6 ¢/h for 25 °C, 30 °C and 35 °C. Another advantage of the system is that hot water is obtained at set temperatures. In PV/T-VCRS, 475 L, 300 L, and 210 L of hot water were obtained at 25 °C, 30 °C, and 35 °C, respectively. As a result, the performance of the PV/T-VCRS was good, with the added benefit of performing close to the performances when PV/T and VCRS were used separately.

## Highlights

• PV/T-VCRS was evaluated in different conditions experimentally in this study.
• 8.5 % lower surface temperature of the module in PV/T-VCRS 25 °C was measured compared to PV/T-VCRS 30 °C and 35 °C.
• In direct proportion to the module surface temperatures, 13 % better electrical efficiency was obtained in PV/T-VCRS 25 °C compared to 30 °C and 35 °C.
• The COP value increased by 15.46 % in PV/T-VCRS 25 °C compared to 30 °C and 35 °C.

## 1 Introduction

Technological development and economic growth in the world depend on energy. In recent years, an enhancement in energy demand has been observed because of population growth, economic problems, and per capita energy consumption [1,2]. A large part of the energy need in the globe is met by fossil fuels [3]. However, fossil fuel sources are energy sources that cause high rates of global warming and greenhouse gases. The ever-increasing energy consumption on a global scale has led to the need to use energy resources more efficiently [4].

Buildings are an area with the most important and suitable potential for energy saving. However, while space heating and hot water production in residences account for 80 % of the energy claim, the energy claim for cooling is increasing day by day. Vapor compression refrigeration system (VCRS) is widely utilized in refrigeration systems [5,6]. Although VCRSs are efficient systems, synthetic refrigerants such as hydrochlorofluorocarbons (HCFC), hydrofluorocarbons (HFC), and chlorofluorocarbons (CFC) are generally preferred in the use of refrigerants. They deplete the ozone layer and induce the greenhouse impact when these refrigerants are handed out into the atmosphere [7].

In recent years, cognizance of global warming has been established and the investigation for alternative energy sources that are detached from fossil fuels and that may cause less global warming has been revived [8]. It is aimed at decreasing the world's energy dependence on fossil fuels, diversifying resources and controlling energy consumption [9]. Interest in renewable energy is constantly increasing, as they have important potential to contribute to sustainable improvement [10]. Solar energy stands out among renewable energy sources because it is clean and endless.

Also, one solution to address the increase in climate change and global energy demand will be to use renewable energy to provide refrigeration alternatively fossil fuel-devouring air conditioning systems, doing solar refrigeration system applications significant for the forthcoming [11]. Solar energy is presently a topic of refrigeration and major interest is especially interesting enforcement because of the overlap between the summit of refrigeration claim and the availability of solar radiation [12].

Solar systems are suitable for both heating and refrigeration [13,14]. Basic enforcements for solar energy technologies; applications that suppose low-temperature heat such as pool heating, drying process, space heating, industrial processes, domestic water heating, and agriculture. Relying on other energy utilizes in residences, domestic water heating can represent 30 % of the energy consumed [15,16]. Today, solar water heaters exemplify one of the most effective solar energy applications. They make up the batch of the present solar heating and refrigeration market, producing approximately four times more energy than all solar technologies combined [17,18].

Solar applications have the potential to meet refrigeration demands with the advantage that procurement and claim are well paired. Combined systems can be used comfortably to create a synergy effect in climates that need heating and refrigeration.

There are many studies on solar energy and refrigeration systems, both separately and in combination. Abdullah et al. [19] developed and studied a novel absorber copper tube flow designed based on a PV/T module with water. The simulation outcomes obtained were compared with the experimental outcomes. Water-based PV/T module performances for thermal, electrical, and overall efficiency are determined in diverse flow rates between 2 and 6 l/min and the solar radiation change of 500–1000 W/m^2^. Maximum thermal efficiency is determined as 58.64 % in 5 l/min and 1000 W/m^2^, maximum electrical efficiency was 11.5 % in 6 l/min and 500 W/m^2^, and total efficiency was determined as 66.87 % in 5 l/min and 1000 W/m^2^. As a result, it was shown that the enhancement in solar irradiance and water flow rate brings out an enhancement in overall and thermal efficiency, but after the optimum circumstance, the overall and thermal efficiency reduces. The increased water flow rate decreased the module temperature and increased the electrical efficiency [19]. Dannemand et al. [20] examined a PV/T module-supported heat pump system with a hot water tank to obtain domestic hot water and a cold water tank around the heat pump's energy source. The experimental setup was analyzed by the measurements made for nine months. During the sunny summer periods, almost all of the hot water needs are met by the PV/T module's thermal part. During less sunny days, the heat pump refilled the dwindling hot water tank. During less sunny and colder periods, the PV/T module provides an important quantity of energy to the cold water tank [20]. Zarei et al. [21] aimed to investigate a heating/refrigeration system's performance utilizing a PV/T module for residential applications. Water was utilized to refrigerate the module and thus enhance its efficiency in this study. Different two refrigerants (R290 and R600a) which have low global warming potential were utilized alternatively to R134a to diminish the global warming effect. The results are that at 945 W/m^2^ solar irradiance and enhancing the water mass flow rate from 0.011 kg/s to 0.03 kg/s, the PV/T module's overall efficiency is 75 % from 66.7 %. In the way of the lowest exergy loss and the highest COP, R290 outperformed other refrigerants [21]. Zhou et al. [22] examined a single-stage compression PV/T module-supported heat pump system. The designed system consists of four PV/T modules, a 0.745 kW heat pump, a 150 L water tank, and numerous monitoring sensors to see the operating circumstances. The test outcomes displayed that the power and PV module's electrical efficiency were 286 W and 11.8 %, respectively. Thermal efficiency, heating power, and COP were calculated on average as 120 %, 4.7 kW, and 6.16 %, respectively. In general, it took approximately 70 min to raise the temperature of 150 L water to 35 °C [22]. Alomar and Ali [23] analyzed in detail the effects of operating circumstances and climate on the PV/T module's electrical and thermal efficiencies. Energy and exergy analyses of the PV/T module were made to analyze the system performance. Experimental data were obtained because of measurements made on three different days. It was determined climatic circumstances have an important impact on exergy and energy outputs. According to the energy analysis outcomes, the maximum electrical and thermal efficiencies changed between 12-16 % and 25–58 %, respectively. In accordance with the exergy analysis, optimum electrical and thermal efficiencies were observed in the changes of 2–7% and 10–18 %, respectively [23]. Mi et al. [24] examined the shortcomings of the unlike hot water supply systems with multiple heat sources and analyzed the PV/T module-supported heat pump's performance. Here, an application is made to reduce the system's cost and enhance its efficiency. As a result of the analysis, daily hot water production is enhanced by 80 %. The system's economic performance was developed by 49 % [24]. Abdul-Ganiyu et al. [25] evaluated the impacts of distinct mass flow rates on a PV/T module's performance in Ghana. A water-based flat plate PV/T module and the PV module are located adjoining on a ceiling. The electrical, thermal, and exergy performances of the PV/T module were investigated in mass flow rates between 0.025 and 0.083 kg/s. No important enhancement in cell temperature was observed when the mass flow rate exceeded 0.082 kg/s for a given solar radiation. The PV/T module showed a constant exergy efficiency of around 12.75 % in the manufacturer's proposed mass flow rate of 0.033 kg/s, regardless of radiation. However, the PV module's exergy efficiency is lower for radiations below 790 W/m^2^. Although the PV/T module's efficiency was commonly at 50 %, the thermal efficiency of 38.8–43.1 % was achieved [25]. Aghakhani et al. [26] modeled solar panels' cooling utilizing copper pipes. Variables used in the system include tube diameters of 9.53, 12.70, and 15.88 mm and water flow rates in the change of 0.5–2.5 l/min. Finally, the simulation program was optimized to acquire the best working circumstances. Consequently, the highest electrical efficiency (14.8 %) is acquired with a flow rate of 2.5 l/min and a pipe diameter of 9.53 mm [26]. Abdul Ganiyu et al. [27] performed the economic and technical analysis of monocrystalline PV module and water-based monocrystalline PV/T module established in Ghana. Some models have been improved to make economic and technical analyses of the PV and PV/T modules over a period of 25 years. Consequently, the predicted average annual total exergy from the PV/T and PV module was obtained as 330.15 kWh/m^2^ and 159.42 kWh/m^2^. As a result of the economic analysis, it is 0.33 $/kWh and 0.45 $/kWh, respectively, for 4.6 h of peak sun time at the installation site of the PV/T and PV module. If the average peak sun time is increased from 4.6 h to 6.5 h, 18 % and 11 % reductions in the economic values of the PV and PV/T modules were appointed [27]. Del Amo et al. [28] analyzed an optimization analysis of the system by integrating a solar-powered water-to-water heat pump system at the university building in Spain. Starting from the present design of the experimental setup, discrete circumstances were simulated to examine different forms of the heating system. The predicted payback period is 15.4 years. Three different forms were suggested, accomplishing 98 % of solar coverage. In the economic analysis, the lowest unit cost of 0.72 €/kWh was obtained in the circumstance three configurations [28]. Marzouk et al. [29] investigated the air bubble injection method to increase heat transfer in tubular heat exchangers. In the study, air was injected into the pipe sides and the waterside flow rate was kept constant at 18 l/min. Two different types of configurations were tested. In performance parameters, configuration A increased by 33%–143 %, and configuration B increased by 44%–184 %. Additionally, air injection had less than 5 % impact on heat exchanger performance parameters [29]. Marzouk et al. [30] used six different deflectors to improve the thermal performance of shell and tube heat exchangers. Baffle configurations include single segment, hybrid segment, stepped single segment, flower segment, circular ring segment, and perforated circular ring segment. When the maximum values of the efficiency and heat transfer coefficient of the shell heat exchanger were compared with the single segment baffle, an improvement of 166 % and 142 % was found in the perforated annular ring, respectively [30]. Marzouk et al. [31] tested the fractal configuration in coiled tube heat exchangers and compared their performance. According to the results, it was observed that the fractal tube heat exchanger had better performance in heat transfer than the standard spiral tube heat exchanger. The heat transfer coefficient improvement rate was determined to be approximately 289 %. The high exergy efficiency in the fractal tube heat exchanger increased with the increase of Reynolds number. The fractal tube heat exchanger provided a pressure reduction of approximately 57 %. This significantly reduced pumping power [31]. Marzouk et al. [32] investigated the thermal, hydraulic and thermodynamic performances by adding a shell with wire-studded circular cut bar inserts to the tube side of the heat exchanger. While the hot water pipe side flow rate varies between 13 and 18 l/min, a water flow rate fixed at 18 l/min was taken into account on the kabuki side. Compared to the conventional design, heat transfer coefficient, efficiency and exergy efficiency were improved by 210–280 %, 185–224 % and 130–210 % respectively [32]. Al-darraji et al. [33] tested the heat exchanger performance by experimenting with single segment, disk, and ring and circular perforated disk and ring baffle configurations. As a result of the experiments, the circular hole disc and ring configuration provided a 231 % increase in the heat transfer coefficient [33].

Most of the above-mentioned studies were tested separately to improve the performance of PV/T or heat exchangers. In this study, PV/T and VCRS were used together and analyzed thermodynamic, thermoeconomic and enviroeconomic at different water tank temperatures (25 °C, 30 °C, and 35 °C) to improve the performance of both components. The most important contribution to the literature in the study is superheating. In conventional applications, superheating is done by locating a heat exchanger in the outlet of the evaporator and the outlet of the condenser. Thus, liquid refrigerant is prevented from entering the compressor. In this way, the compressor is not damaged. In this study, out of this conventional application, superheating is done with a PV/T module. The heat exchanger in the PV/T-VCRS exposes the evaporator outlet and the cooling water from the module to heat transfer. A heat transfer is ensured to the refrigerant, which is colder than water, thus preventing the liquid refrigerant from going to the compressor. The cooling water used in the solar module is passed behind module and the PV module's temperature is kept at low levels. Thus, the PV/T module's electrical efficiency is enhanced. The PV/T-VCRS's other advantage is that the electricity produced in the PV/T module provides the electrical energy needed by the evaporator fan, pump, and control devices in the system.

## 2 Material and methods

The properties of the materials utilized in the study and the theoretical expressions of the methods used are included in this section. Thermodynamic analyses are used to examine the PV/T-VCRS's performance. Thermoeconomic analysis was applied for the cost analysis of the system and enviroeconomic analysis was applied to analyze the PV/T-VCRS from the environmental view.

### 2.1 Operation of the experimental system

**Fig. 1:**
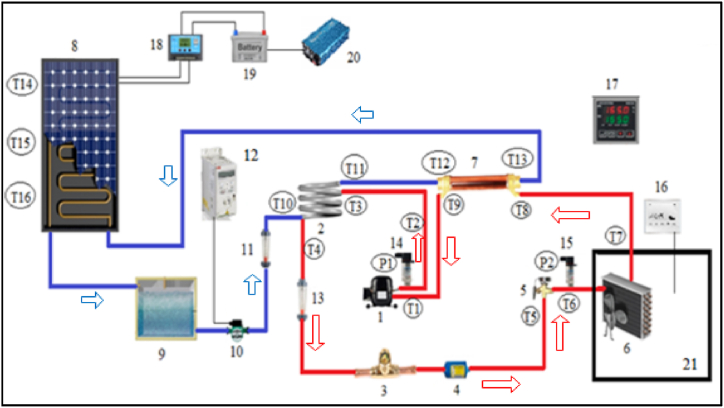
General view of the PV/T-VCRS.

**Fig. 2:**
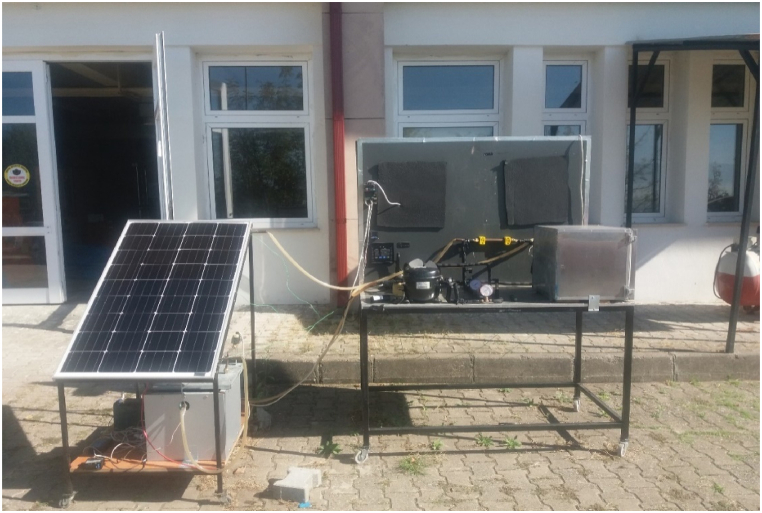
View of the experimental setup.

In this experimental study, VCRS is assisted by a PV/T module. The experimental system comprises two parts, the PV/T module and VCRS. The general view of the PV/T-VCRS is indicated in Fig. 1 and view of experimental setup in Fig. 2. In the VCRS, R134a was utilized as a refrigerant. Generally, liquid refrigerant can be found in the refrigerant vapor sent from the evaporator to the compressor at low ambient temperatures. Liquid refrigerant flowing into the compressor poses a great danger to the compressor and seriously reduces the compressor's working life. In order to eliminate this problem that may occur in the compressor, superheating is applied in the refrigeration systems. In conventional applications, heat transfer is carried out from refrigerants at different temperatures by locating a heat exchanger in the evaporator and condenser outlets. In this study, unlike traditional applications, superheating was applied with the help of a PV/T module. Since the temperature of the water coming from the PV/T module is higher than the evaporator temperature, a heat transfer takes place from the water to the refrigerant. The internal heat exchanger is located between the evaporator and the compressor in PVT/VCRS. The superheating is realized by exposing the refrigerant coming out of the evaporator and the water coming from the PV/T module with the help of the circulation pump to heat transfer. The flow type in the double pipe condenser and the internal heat exchanger is counterflow. The PV/T- VCRS's other advantage is that the water, which loses its heat and cools for the superheating process, is sent back to the PV/T module, so increasing the electrical efficiency by reducing the module's surface temperature. PV modules are examined at the module's temperature of 25 °C pending manufacturing, and their electrical efficiency is calculated with respect to this temperature. According to the literature, it was determined that each 1 °C enhancement in the module temperature decreases cell efficiency by 0.45 % [34,35]. Because of this, lowering the module temperature enhances the module's electrical efficiency. In this study, the module temperature is lowered because of the water's cooling in the superheating going back to the module. Thus, the module's electrical efficiency is enhanced with the decrease in the module temperature.

The experiments were carried out outdoors between 11:10 and 17:30 in July. The data were generally taken on days when the weather was clear and cloudless.

The water distributed back of the PV module in the PV/T module's thermal part is warmed up and sent to the water tank. The water in the tank is sent to the condenser with double pipes in the VCRS. The set temperatures (25 °C, 30 °C, and 35 °C) were determined to control the water's temperature. The solenoid valve that controls the mains water connected to the tank opens with the signal received from the thermostat when the water tank's temperature rises above the set temperature. Mains water is delivered to the tank until the water tank temperature is reduced to the set temperature. The water's circulation in the PV/T module is supplied with the help of the pump. Technical properties of PV/T-VCRS is given in Table 1.

**Table 1:** Technical properties of the components utilized in PV/T-VCRS.

Component	Technical specifications
PV Module	Maximum power: 150 W
	Open circuit voltage: 22.80 V
	Short circuit current: 6.98 A
	Dimensions: 1150x675x28 mm
	Cell type: Monocrystalline
Compressor	Power: 3/8 HP
	Refrigerant: R134a
	Cooling capacity: 325–920 kcal/h
Fan	S&P TD-250/100, 230–240 V, 50–60 Hz, 24 W
Condenser	Capacity: 1 kW
Thermostatic expansion valve	Temperature range: 40/10 °C
Drier (Filter)	Temperature range: 40/70 °C
	Net volume: 0.464 l
	Maximum operating pressure: 46 bar
Water tank	Volume: 30 l
Pump	Flow rate: 2–6 l/min
Battery	12 V, 65 Ah
Solar charge regulator	MPPT Charge control device, Charge current: 10 A

### 2.2 Installation of the experimental system

**Fig. 3:**
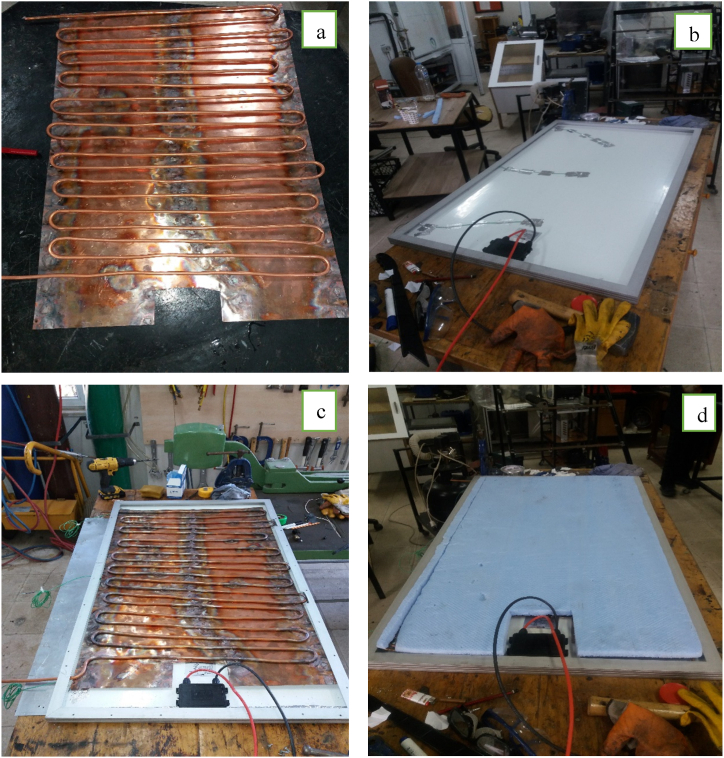
Stages of the PV/T module design a) Designed heat exchanger, b) View of thermocouples placed in 3 different places behind the PV c) Placement of the designed heat exchanger behind the PV d).

The PV/T module used in the experimental system was made manually. A heat exchanger is designed to pass cooling water behind the supplied PV module. This heat exchanger consists of a 3/8″ diameter copper pipe. A 0.5 mm thick copper sheet was added to the heat exchanger surface to increase heat transfer. K-type thermocouples are located at three discrete points behind the PV module to determine the module temperature. While calculating the module temperature, the average of these three different points was taken. After this process, the heat exchanger, which is combined with a copper plate, was located on the PV module's back. Then, 5 mm thickness, Extruded Polystyrene (XPS) insulation material was placed on the heat exchanger so that the PV/T module would not enter into the heat exchanger with the environment. Eventually, the back of the PV/T module is covered with 1 mm thick sheet metal. The PV/T module's angle with the horizontal is 41°. The PV/T module's manufacturing stages are given in Fig. 3.

The second part of experimental system is the VCRS with a heat exchanger. The refrigeration system components (compressor, condenser with double pipes, evaporator, expansion valve, etc.) whose locations have been determined have been conveniently placed and fixed. Then, the connections of each of the system components were welded together with copper pipes. After the entire welding process was completed, a leakage test was carried out by introducing dry nitrogen into the system. After it was understood that there was no leakage in the system, the system was vacuumed. After the vacuum operation, 200 g of R134a refrigerant was charged.

The control system is of great importance for the test system to operate more efficiently and reliably. There are two different control mechanisms in the system. One of them is used to change the cooling water's velocity provided to the system and to prevent the compressor from getting too hot. The other control mechanism is used to keep the water tank's temperature at the set temperature. In the first control system, the compressor outlet temperature is determined with a thermocouple joined to the controller. The compressor outlet temperature is set to 40 °C. If the compressor outlet temperature is lower than the set temperature, the frequency inverter sends a signal to the circulation pump to operate at 30 Hz. When the compressor outlet temperature rises above the set temperature, the frequency inverter sends a signal to increase the speed to 50 Hz for the circulation pump to send more cooling water. Thus, the compressor's temperature is prevented from rising to very high values. In the other control mechanism, the change in system performance was followed by adjusting the set value so that the condenser could release more heat. Here, the experimental conditions of 25 °C, 30 °C, and 35 °C were used as the water tank's temperature. When the cooling water from the PV/T module comes to the water tank, it arrives at high temperatures. The hot water that goes to the condenser at a high temperature from here prevents the condenser from throwing more heat than normal and in this circumstance reduces the system's performance. This control system was used to control it. While the thermostat controls the water tank's temperature from this control system, a solenoid valve is located at the entrance of the mains water line entering the water tank. The solenoid valve and the discharge line pump are closed when the tank temperature is below the set value. When the water tank's temperature enhances above the set value, the thermostat sends a signal and opens the solenoid valve and the discharge line. With the solenoid valve allowing the mains line to pass, the mains water enters the water tank, and hot water is sent to a different tank to be utilized from the discharge line. In this way, the water temperature going to the condenser is controlled. Thanks to this control system used here, overheating of the condenser is prevented. Because it is undesirable for the double pipe heat exchanger used in the experiment system to reach extreme temperatures. There is a danger of swelling when the condenser reaches extremely high temperatures. Therefore, it is desirable for the condenser to dissipate heat better for system safety. In addition, better heat dissipation of the condenser is positive for both superheating and system performance.

### 2.3 Thermodynamic analysis

Thermodynamic analysis is a commonly used technique to specify the performance of thermal systems. This analysis consists of two analyses energy and exergy analysis. Energy analysis is accomplished using thermodynamics' fundamental laws to analyze a system's operating performance [[36], [37], [38]]. Thermodynamics' first law is the law of energy conservation and is concerned only with the amount of energy. In general, thermodynamics' first law is considered in the energy analysis of a system [39,40].

Exergy analysis is applied according to the thermodynamics' second law. Exergy analysis is a method that utilizes the principles of mass conservation and energy conservation, together with thermodynamics' second law for the design, analysis, and development of energy and other systems. Consequently, the results of the exergy analysis are made according to the specified reference medium environment. A system's exergy in equilibrium with the reference environment is zero [39].

#### 2.3.1 Analysis of VCRS

Thermodynamic analysis of the VCRS is applied in this section. The VCRS's performance analysis was applied according to thermodynamics' first and second laws. The components of VCRS are determined in Equations (1), (2), (3), (4)):

(1) $${\dot{Q}}_{evap}=\dot{m}\left(h_{7}-h_{6}\right)$$

(2) $${\dot{Q}}_{cond}=\dot{m}\left(h_{4}-h_{3}\right)$$

(3) $${\dot{W}}_{comp}=\dot{m}\left(h_{2}-h_{1}\right)$$

(4) $${\dot{W}}_{comp,el}=\frac{{\dot{W}}_{comp}}{\eta_{mech}\times \eta_{el}}$$

The COP of the VCRS corresponds to the ratio of the compressor work to the absorbed heat from the environment by the evaporator. COP is calculated in Equation 5

(5) $$COP=\frac{{\dot{Q}}_{evap}}{{\dot{W}}_{comp,el}}$$

Exergy analysis is significant to settle the asset of energy in the VCRS. The exergy destruction for a continuously flowing control volume is expressed in Equation (6).

(6) $${\dot{Ex}}_{destr}=\sum {\dot{Ex}}_{in}-{\dot{Ex}}_{out}+\sum {\left[\dot{Q}\left(1-\frac{T_{0}}{T}\right)\right]}_{in}-\sum {\left[\dot{Q}\left(1-\frac{T_{0}}{T}\right)\right]}_{out}+\sum {\dot{W}}_{in}-\sum {\dot{W}}_{out}$$

The flow exergy for the apiece cycle realized in the VCRS is determined in Equation (7).

(7) $$\dot{Ex=}\dot{m}\left[h-h_{0}-T_{0}\left(s-s_{0}\right)\right]$$

The examined system comprises five components a condenser, compressor, evaporator, etc. The exergy losses in these components are given in Equations (8), (9), (10), (11), (12), (13), (14), (15), (16), (17)). The exergy loss for the compressor is shown in Equation (8) and Equation (9).

(8) $${\dot{Ex}}_{comp\;destr}={\dot{Ex}}_{1}-{\dot{Ex}}_{2}+{\dot{W}}_{comp\;el}$$

(9) $${\dot{Ex}}_{comp\;destr}=\dot{m}\left[\left(h_{1}-T_{0}s_{1}\right)-\left(h_{2}-T_{0}s_{2}\right)\right]+{\dot{W}}_{comp\;el}$$

The general expression of exergy destruction for the condenser is shown in Equation (10) and Equation (11).

(10) $${\dot{Ex}}_{cond\;destr}={\dot{Ex}}_{3}-{\dot{Ex}}_{4}-\left[{\dot{Q}}_{cond}\left(1-\frac{T_{0}}{T_{cond}}\right)\right]$$

(11) $${\dot{Ex}}_{cond\;destr}=\dot{m}\left[\left(h_{3}-T_{0}s_{3}\right)-\left(h_{4}-T_{0}s_{4}\right)\right]-\left[{\dot{Q}}_{cond}\left(1-\frac{T_{0}}{T_{cond}}\right)\right]$$

Exergy destruction for the evaporator is calculated in Equation (12) and Equation (13).

(12) $${\dot{Ex}}_{evap}={\dot{Ex}}_{6}-{\dot{Ex}}_{7}+\left[{\dot{Q}}_{evap}\left(1-\frac{T_{0}}{T_{evap}}\right)\right]$$

(13) $${\dot{Ex}}_{evap.\;dest.}=\dot{m}\left[\left(h_{6}-T_{0}s_{6}\right)-\left(h_{7}-T_{0}s_{7}\right)\right]-\left[{\dot{Q}}_{evap.}\left(1-\frac{T_{0}}{T_{evap.}}\right)\right]$$

The exergy destruction of the thermostatic expansion valve is calculated in Equations (14), (15)).

(14) $${\dot{Ex}}_{expv\;destr}={\dot{Ex}}_{5}-{\dot{Ex}}_{6}$$

(15) $${\dot{Ex}}_{exvp\;destr}=\dot{m}T_{0}\left(s_{6}-s_{5}\right)$$

The exergy destruction of the internal heat exchanger in the system is shown in Equations (16), (17)).

(16) $${\dot{Ex}}_{hex\;destr}=\left({\dot{Ex}}_{8}-{\dot{Ex}}_{9}\right)+\left({\dot{Ex}}_{12}-{\dot{Ex}}_{13}\right)$$

(17) $${\dot{Ex}}_{hex\;destr}=\dot{m}\left[\left[\left(h_{8}-T_{0}s_{8}\right)-\left(h_{9}-T_{0}s_{9}\right)\right]+\left[\left(h_{12}-T_{0}s_{12}\right)-\left(h_{13}-T_{0}s_{13}\right)\right]\right]$$

The total exergy destruction in the VCRS is determined for system components in Equation (18).

(18) $${\dot{Ex}}_{destr\;total}={\dot{Ex}}_{destr\;comp}+{\dot{Ex}}_{destr\;cond}+{\dot{Ex}}_{destr\;evap}+{\dot{Ex}}_{destr\;expv}+{\dot{Ex}}_{destr\;hex}$$

VCRS's exergy efficiency is shown in Equation (19).

(19) $$\eta_{ex}=\frac{{\dot{Ex}}_{6}-{\dot{Ex}}_{7}}{{\dot{W}}_{comp\;el}}$$

#### 2.3.2 Analysis of PV/T module

Part of the solar energy coming to the module surface is transformed into electrical energy, and the rest to heat energy in the PV/T module. This heat energy influences the electrical conversion efficiency of the module. The PV/T module's electrical energy reduces, as the module's surface temperature enhances. It is supposed that the solar energy coming to the PV/T module is transformed into electrical energy and the part other than the part spent on thermal losses is transferred to the water through copper pipes. The heat energy's quantity converted into electrical energy and transferred to the water inside the copper pipes after whole heat losses are subtracted is given in Equation (20) as follows:

(20) $$\dot{Q}={\dot{m}}_{wat}c_{p,wat}\left(T_{out}-T_{in}\right)$$

PV/T module's filling factor (FF) is calculated by dividing the generation of the maximum current and voltage it can produce by the generation of the module's short circuit current and open circuit voltage. The filling factor calculation is given in Equation (21). The module's output power is given in Equation (22). The PV/T module's electrical efficiency ($\eta_{pv}$) in Equation (23) and the PV/T module's thermal efficiency ($\eta_{th}$) in Equation (24) are as follows, respectively. The PV/T module's total efficiency $\eta_{pvt}$ is seen in Equation (25) as the total of thermal and electrical efficiency.

(21) $$FF=\left(V_{mp}\times I_{mp}\right)/\left(V_{oc}\times I_{sc}\right)$$

(22) $$P_{out}=V_{mp}\times I_{mp}=V_{oc}\times I_{sc}\times FF$$

(23) $$\eta_{pv}=\frac{P_{out}}{A_{m}I_{s}}$$

(24) $$\eta_{th}=\frac{\dot{Q}}{A_{m}I_{s}}$$

(25) $$\eta_{pvt}=\eta_{pv}+\eta_{th}$$

The PV/T module's exergy analysis is based on thermodynamics' second law and includes the calculation of the total exergy input, exergy output, and exergy destruction. Equation (26) an Equation (27) is used for the PV/T module's exergy analysis.

(26) $$\sum {\dot{E}x}_{in}-\sum {\dot{E}x}_{out}=\sum {\dot{E}x}_{destr}$$

or,

(27) $$\sum {\dot{E}x}_{in}-\sum \left({\dot{E}x}_{th}+{\dot{E}x}_{el}\right)=\sum {\dot{E}x}_{destr}$$

The general exergy equation is given as in Equation (28). The exergy output in this equation is equal to the total electrical and thermal exergy and is shown in the equation [41].

(28) $$\sum {\dot{E}x}_{out}=\sum {\dot{E}x}_{th}+\sum {\dot{E}x}_{el}$$

The system's exergy input is equal to the solar radiation exergy. Radiation exergy is determined based on the sun's surface temperature and the air temperature [42]. Radiation exergy is calculated in Equation (29).

(29) $$\sum {\dot{E}x}_{in}=I_{s}.A_{m}\left[1-\frac{4}{3}\left(\frac{T_{a}}{T_{sun}}\right)+\frac{1}{3}{\left(\frac{T_{a}}{T_{sun}}\right)}^{4}\right]$$

In the equation, $T_{sun}$ is the sun's temperature and $T_{a}$ is the ambient temperature. The sun temperature was seized qua 5777 K [43]. The system's thermal exergy (${\dot{E}x}_{th}$) is able to be described as the heat loss to the environment through the PV module and is given in Equation (30).

(30) $${\dot{E}x}_{th}={\dot{m}}_{wat}.c_{p,wat}\left[\left(T_{out}-T_{in}\right)-\left(T_{a}+273\right)\ln\left(\frac{T_{a}+273}{T_{in}+273}\right)\right]$$

In the equation, ${\dot{m}}_{water}$ defines the water circulating's mass flow rate in the PV/T module, $c_{p,water}$ defines the circulating water's specific heat, $T_{in}$ represents the temperature of the water entering the PV/T module and $T_{out}$ describes the temperature of the water leaving the PV/T module [44,45]. Electrical exergy is determined qua in Equation (31) [39].

(31) $${\dot{E}x}_{el}={\dot{E}}_{net}=V_{oc}\;.I_{sc}\;.\;FF$$

Exergy efficiency is calculated as the ratio of outgoing exergy to incoming exergy qua in Equation (32) [46].

(32) $$\eta_{ex,pvt}=\frac{{\dot{E}x}_{out}}{{\dot{E}x}_{in}}$$

#### 2.3.3 Thermoeconomic analysis

Thermoeconomic analysis is a method based on supporting exergy-based thermodynamic analysis with economic data. It is used to improve and develop the system by evaluating the cost-effectiveness of thermal systems. Since this method is related to exergy analysis, it is also called exergoeconomic analysis. In other words, the thermoeconomic analysis method is a method that helps to understand the relationship between exergy destruction in thermal systems and the amount of cost. Thus, an improvement in the system is aimed.

The annual operating cost (ARC) of the system is given in Equation (33). $\dot{m}$ defines the water and refrigerant mass flow rate for the PV/T-VCRS.

(33) $$ARC=\left(\dot{m}\Delta P/\rho \right)t_{op}.CE$$

The present worth of an annuity is the financial worth of the sum of the annuity at the end of a given period if it were deposited at the start of the annuity at the effective interest rate. The recovery factor (CRF) of the initial investment cost is given in Equation (34) [47].

(34) $$CRF=\frac{i{\left(1+i\right)}^{n}}{{\left(1+i\right)}^{n}-1}$$

In equation (34), n is the experimental system's service life, i is the supposed annual interest. The experimental system's service life is supposed 20 years [48], and the supposed annual interest rate is 10 % [49]. FAC is determined qua in Equation (35) and represents the first annual cost. In the equation, TCI represents the initial investment cost. SFF is calculated in Equation (36).

(35) FAC=CRF×TCI

(36) $$SFF=\frac{i}{{\left(1+i\right)}^{n}-1}$$

ASV is defined as the annual salvage value, SFF represents the sinking fund factor, and SV is the salvage value of PV/T-VCRS in Equation (37) [50]. SV is calculated in Equaiton 38.

(37) ASV=SFF×SV

(38) SV=0.12×TCI

Annual maintenance cost (AMC) has been accepted as 10 % of the initial investment cost. In the equation, AC represents the annual cost [51]. AC is calculated in Equation (39).

(39) AC=FAC+AMC+ARC−ASV

Conventionally, the thermoeconomic coefficient is determined as exergy destruction per unit annual cost to diminish loss. Therefore, the thermoeconomic parameter of the system is calculated as [49]:

(40) $$R_{ex}=\frac{{\dot{Ex}}_{out}}{AC}$$

#### 2.3.4 Environeconomic analysis

Fossil fuels, which are mainly used today, enhance the amount of CO_2_ discharged into the environment. Because of the enhancing amount of CO_2_ in the atmosphere, problems such as environmental pollution and global warming arise. Many countries take different measures to figure out these problems and reduce CO_2_ emissions globally. The environmental cost analysis carried out for this purpose is ground on the CO_2_ emission price. The amount of CO_2_ emission decreasing achieved by utilizing the PV/T module-assisted VCRS in different circumstances is given in Equation (41) [52].

(41) $$\varphi_{{CO}_{2}}=\Psi_{{CO}_{2}}.{\dot{Q}}_{total}$$

Here, $\varphi_{{CO}_{2}}$ is the amount of CO_2_ emission that the system reduces, $\Psi_{{CO}_{2}}$ is the CO_2_ value released by the run of coal power plants. $\Psi_{{CO}_{2}}$ value is preferred as 2.08 kgCO_2_/kWh [53,54]. The system's environmental cost value is given in Equation (42).

(42) $$Z_{{CO}_{2}}=z_{{CO}_{2}}.\varphi_{{CO}_{2}}$$

In the equation, $z_{{CO}_{2}}$ is the global carbon price and changes from 13 $/tCO_2_ to 16 $/tCO_2_. $z_{{CO}_{2}}$ is preferred as 14.5 $/tCO_2_ in the equations [55,56].

#### 2.3.5 Uncertainty analysis

Uncertainty analysis finds out the results' accuracy limit obtained from experiments in an experimental study and helps with how accuracy is obtained in experimental studies. Uncertainties were obtained utilizing Equation (43), (44), (45), (46)), taking into account the measuring devices’ standard deviations utilized in the PV/T-VCRS [46,57].

(43) $$X_{m}=\frac{1}{N}\sum X_{i}$$

(44) $$S={\left[\frac{1}{\left(N-1\right)}\sum_{i=1}^{N}{\left(X_{i}-X_{m}\right)}^{2}\right]}^{1/2}$$

(45) $$a=\frac{1}{\sqrt{N}}$$

(46) $$U=\sqrt{\sum_{i=1}^{R}{a_{i}}^{2}.{S_{i}}^{2}}$$

$X_{m}$ defines the experimental measurements' arithmetic mean in Equation (43). $X_{i}$ coincides with the i measurement of the taken coefficient. S represents the standard deviation in Equation (44). a shows sensitivity in Equation (45) and U is uncertainty in Equation (46). The uncertainty worths of the measuring instruments utilized in the PV/T-VCRS are indicated in Table 2.

**Table 2:** Technical properties of the measurement instruments utilized in the PV/T-VCRS.

Measurement instruments	Technical specifications	Uncertainties
Flowmeter	Measuring range: 0–16 g/s, Accuracy: 1 g/s	± 0.03 g/s
Powermeter	Measuring range: 0.1–3680 W, Accuracy: 0.1 W	± 0.32 W
Thermocouple	K Type	± 0.45 °C
	Measuring range: 30/130 °C, Accuracy: 0.5 °C	
Solar meter	Measuring range: 0–2000 W/m^2^	± 8.03 W/m^2^
	Accuracy: ±10 W/m^2^ or ±5 %	
Thermostat	NTC: 40/+105 °C,	± 0.365 °C
	Relay output: 1 piece, 16 A, Digital input: 1	
Pressure transmitter	Measuring range: 0–30 bar, Accuracy:1 bar	± 0.2 bar

## 3 Results and discussion

In this study, energy, exergy, thermoeconomic, and enviro-economic analyses of the hybrid system are interpreted. The designed system is examined in three different circumstances the PV module, PV/T module, and PV/T-VCRS. In addition, the PV/T-VCRS is tested by setting it at different temperatures in the water tank (25 °C, 30 °C, and 35 °C). Data from the experimental system were taken every 10 min. Measurements were taken from three diverse points and the average of these worths was taken to determine the module temperature.

**Fig. 4:**
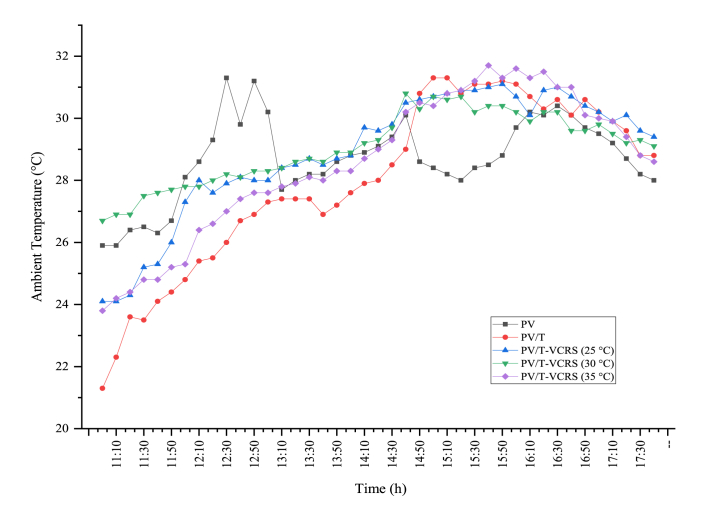
Changes in ambient temperatures in experiments performed in different circumstances.

In the experiments conducted to evaluate the system performance, since the data were taken on different days, the similarity of the temperatures does not seriously affect the accuracy of the experimental results. The changes in ambient temperatures while data were being taken in the experiments are given in Fig. 4. The average ambient temperature at the PV module was measured as 28.68 °C, the average ambient temperature at the PV/T module was measured as 27.98 °C, the average ambient temperature at the PV/T-VCRS (25 °C) was measured as 28.88 °C, the average ambient temperature at the PV/T-VCRS (30 °C) was measured as 29.04 °C and the average ambient temperature at the PV/T-VCRS (35 °C) was measured as 28.55 °C. Although different values were measured in ambient temperatures during the day during the experiments, it was observed that the average temperature values were very close to each other. For this reason, it is proof that the ambient temperature, which is one of the parameters used in calculating system performance, is reliable.

**Fig. 5:**
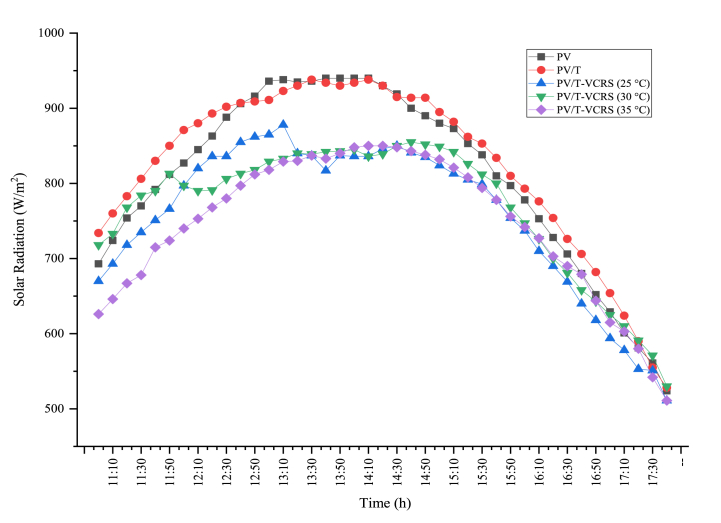
Solar radiation changes measured in experiments.

Since the solar radiation measurements, which affect the system performance as well as the ambient temperature, are taken on different days, the closeness of the experimental conditions will facilitate the meaningful interpretation of the results obtained in the performance calculations. Solar radiation data measured during the experiments are given in Fig. 5. As seen in the figure, it is seen that the data taken on different days are parallel to each other except for minor fluctuations. The daily average solar radiation obtained in PV is 809.22 W/m^2^, the daily average solar radiation obtained in PV/T is 823.41 W/m^2^, the daily average solar radiation obtained in PV/T-VCRS (25 °C) is 758.07 W/m^2^, the daily average solar radiation obtained in PV/T-VCRS (30 °C) is 768.37 W/m^2^ and the daily average solar radiation obtained in PV/T-VCRS (35 °C) is 746.22 W/m^2^. Therefore, it shows that the comments will be meaningful when the performance results are obtained.

**Fig. 6:**
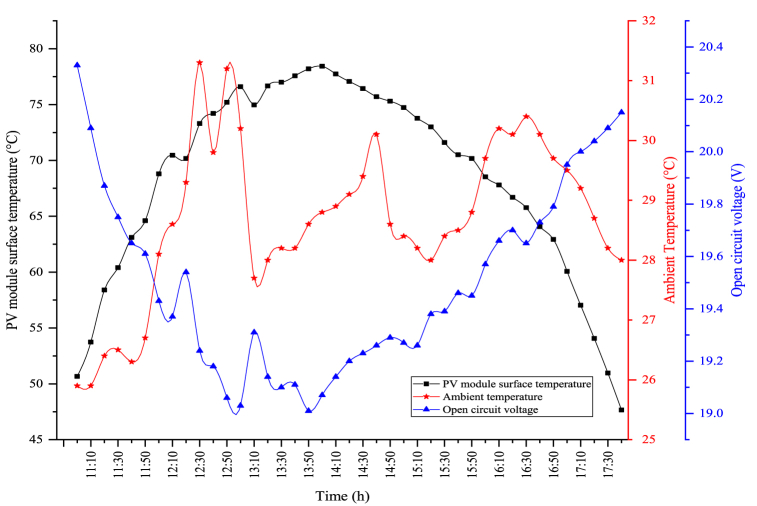
Changes in the module temperature and open circuit voltage.

The module temperature plays an important role in the electrical efficiency of PV modules. The surface temperature and open circuit voltage changes of the module are indicated in Fig. 6. The maximum module temperature is observed as 78.43 °C when the solar radiation is the highest in the module. The average module temperature was calculated as 68.39 °C. The average open circuit voltage was determined as 19.50 V during the experiment period, while the maximum open circuit voltage in the PV module was determined as 20.33 V. As the module temperature increases, the open circuit voltage reduces, and as the module temperature reduces, the open circuit voltage enhances.

**Fig. 7:**
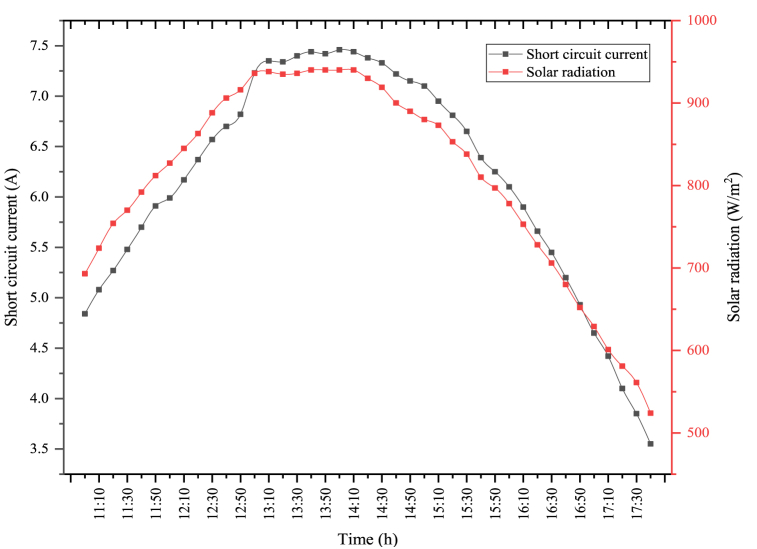
Changes in solar radiation and short circuit current of PV module.

The short circuit current and the solar radiation changes obtained when the PV module is used alone are indicated in Fig. 7. In the figure, the maximum solar radiation was determined as 940 W/m^2^. The average solar radiation was determined as 809.22 W/m^2^. The solar radiation coming to the module directly affects the short circuit current obtained from the module. In general, solar radiation and short circuit current are parallel to each other. As seen in the figure, the maximum short circuit current was measured as 7.46 A, where the solar radiation was high. The average short circuit current was measured as 6.17 A. Short circuit current generated in the module directly affects the PV module's power, which in turn affects the PV module's electrical efficiency.

**Fig. 8:**
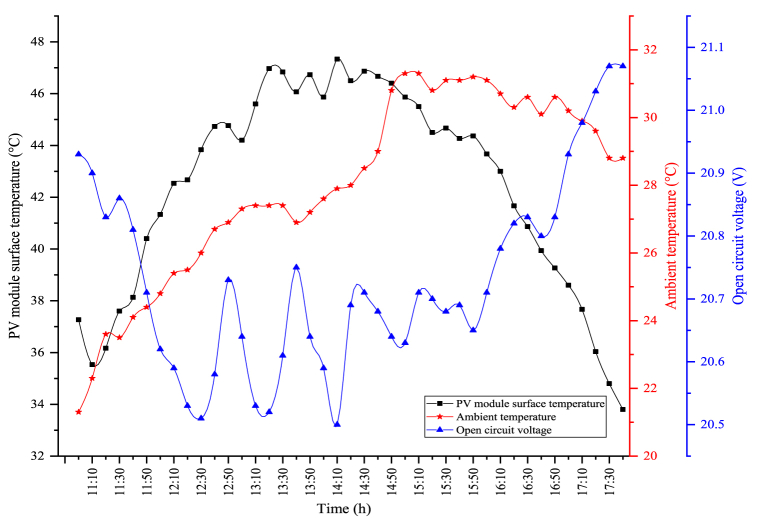
Changes in the module temperature and open circuit voltage of the PV/T module.

The changes in the module temperature and open circuit voltage obtained when the PV/T module is utilized alone are indicated in Fig. 8. In the figure, the module temperature and the open circuit voltage follow each other in parallel. The open circuit voltage decreases when the module temperature increases, and as the module temperature reduces, the generated open circuit voltage is enhanced. The maximum module temperature in PV/T is observed as 47.33 °C. The average module temperature was determined as 42.43 °C throughout the experimental period. The most important factor for the lower module temperature in the PV/T module compared to the circumstance where the module is used alone is the water passing back of the module. The water flowing behind the module reduces the surface temperature. Therefore, as the module temperature decreases, more open circuit voltage is obtained. The fluctuations seen in the figure are caused by the cooling of the module's surface. An average open circuit voltage of 20.73 V was obtained. The maximum open circuit voltage obtained in the PV/T module was observed as 21.07 V.

**Fig. 9:**
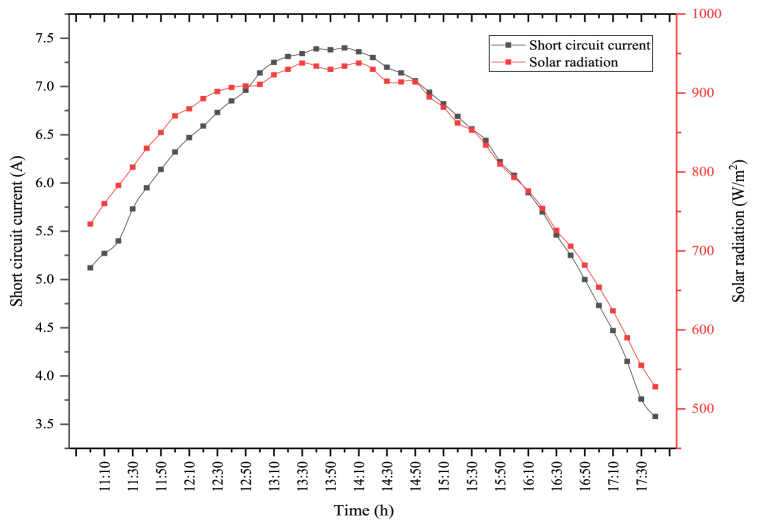
Changes in short circuit current and solar radiation of the PV/T module.

The changes in short circuit current and solar radiation in the PV/T module are indicated in Fig. 9. In the figure, the average amount of solar radiation during the experiment was determined as 823.41 W/m^2^. The maximum solar radiation was obtained as 938 W/m^2^. The maximum short circuit current acquired from the solar radiation coming into the module was measured as 7.39 A. During the experiment period, the average short circuit current was determined as 6.21 A. As in the module, short circuit current and solar radiation are parallel to each other in the PV/T module. As solar radiation enhances, the short circuit current generated enhances, and as the solar radiation reduces the short circuit current decreases. This circumstance directly influences the PV module's electrical efficiency.

**Fig. 10:**
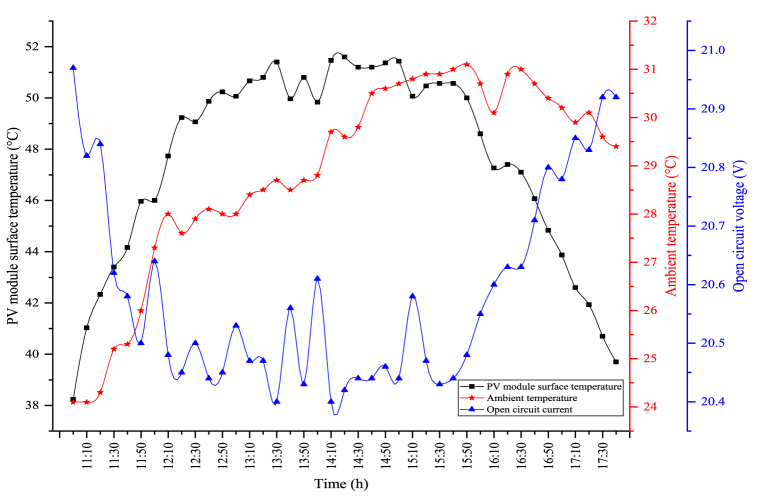
Changes in surface temperature and open circuit voltage of the PV/T-VCRS (25 °C).

The changes in the PV module's surface temperature and open circuit voltage obtained in the hybrid system are indicated in Fig. 10. In the figure, the correlation between open circuit voltage and the PV module's surface temperature shows parallelism. The average surface temperature of the PV module was determined as 47.58 °C during the experiment period. The maximum PV module's surface temperature is measured as 51.6 °C in the PV/T-VCRS (25 °C). The PV module's surface temperature is higher than the PV/T module in the hybrid system. The reason for this is that while the average temperature of the water passing behind the module in the PV/T module is 20 °C, this temperature is higher in the hybrid system. For this reason, the module's surface is less cooled. This leads to a lower open circuit voltage. The average open circuit voltage obtained during the experiment period was determined as 20.58 V. The maximum open circuit voltage obtained in the hybrid system was measured as 20.97 V. In the figure, as the PV module's surface temperature increases, the open circuit voltage decreases, while the open circuit voltage increases as the PV module's surface temperature decreases. This circumstance directly affects the power obtained from the module. The resulting high open circuit voltage increases the power of the PV module, which in turn increases the PV module's electrical efficiency.

**Fig. 11:**
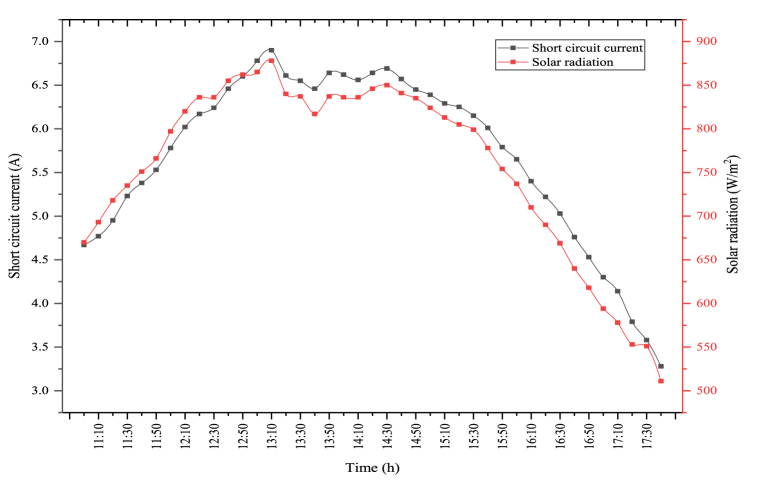
Changes in short circuit current and solar radiation of the PV/T-VCRS (25 °C).

The changes between solar radiation and short circuit current in the hybrid system are indicated in Fig. 11. In the figure, there is a similar change between solar radiation and the short circuit current. The maximum solar radiation was measured as 878 W/m^2^ in the PV/T-VCRS (25 °C). During the experiment, the average solar radiation was obtained as 758.07 W/m^2^. The solar radiation coming to the module is the most significant parameter affecting the produced short circuit current value. The maximum short circuit current and the average short circuit current are determined during the experiment period as 6.9 A and 5.7 A in the hybrid system, respectively. The maximum and average short circuit currents are higher than the PV/T-VCRS (25 °C) when the PV module is tested alone. This circumstance is because the daily direct solar radiation value is different regardless of the system.

**Fig. 12:**
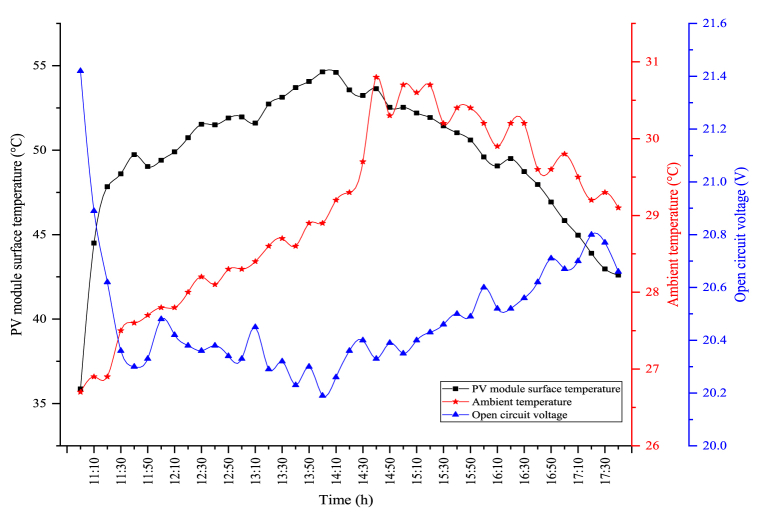
Changes in open circuit voltage and module temperature of the PV/T-VCRS (30 °C).

The changes between the open circuit voltage and the PV module's surface temperature obtained because of the experiment in the PV/T-VCRS (30 °C) are shown in Fig. 12. In the figure, there is an inverse relationship between the PV module's surface temperature and the open circuit voltage. The maximum module temperature obtained in the PV/T-VCRS (30 °C) was obtained as 54.63 °C. During the experiment period, the module temperature is determined as 49.8 °C. The open circuit voltages obtained according to these module temperatures show differences. The module temperature at the set temperature of 30 °C was higher than the set temperature of 25 °C in the PV/T-VCRS. This is because the water temperature passed behind the module is higher. This temperature increase directly affects the open circuit voltage. In this circumstance, it directly influences the power generated by the module and accordingly the electrical efficiency. The maximum open circuit voltage obtained in the PV/T-VCRS (30 °C) was measured as 21.42 V. The average open circuit voltage obtained during the experiment period was determined as 20.49 V. It is seen that the open circuit voltage was lower at 30 °C when the set temperatures of 25 °C and 30 °C are compared with each other in the PV/T-VCRS. This is because the temperature of the water passing behind the module is higher.

**Fig. 13:**
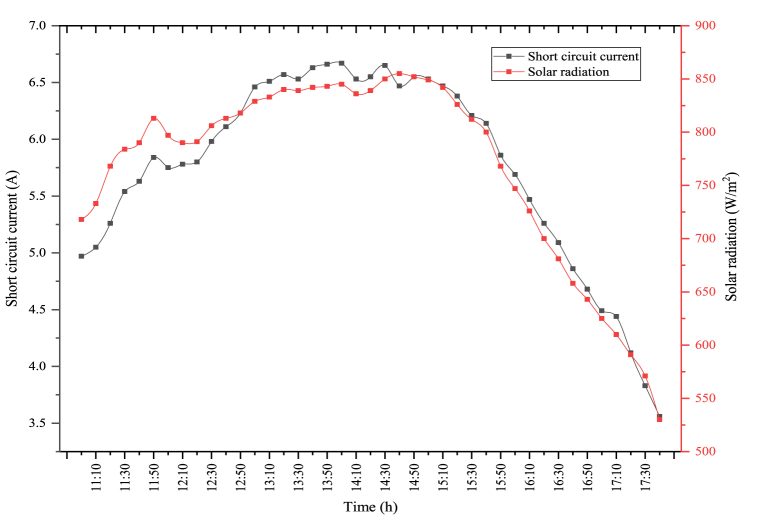
Changes in short circuit current and solar radiation of the PV/T-VCRS (30 °C).

The changes between short circuit current and solar radiation because of the test performed in the PV/T-VCRS (30 °C) are shown in Fig. 13. In the figure, there is a direct connection between short circuit current and solar radiation. The interaction between the two parameters acts similarly to each other. The average solar radiation during the experiment period was determined as 768.37 W/m^2^. Maximum solar radiation in the PV/T-VCRS (30 °C) was measured as 855 W/m^2^. Solar radiation is important in terms of the module power and electrical efficiency. Because the amount of solar radiation coming to the module surface directly influences the amount of short circuit current generated in the module. The maximum short circuit current is measured as 6.67 A in the PV/T-VCRS (30 °C). During the experiment period, the average short circuit current was determined as 5.75 A.

**Fig. 14:**
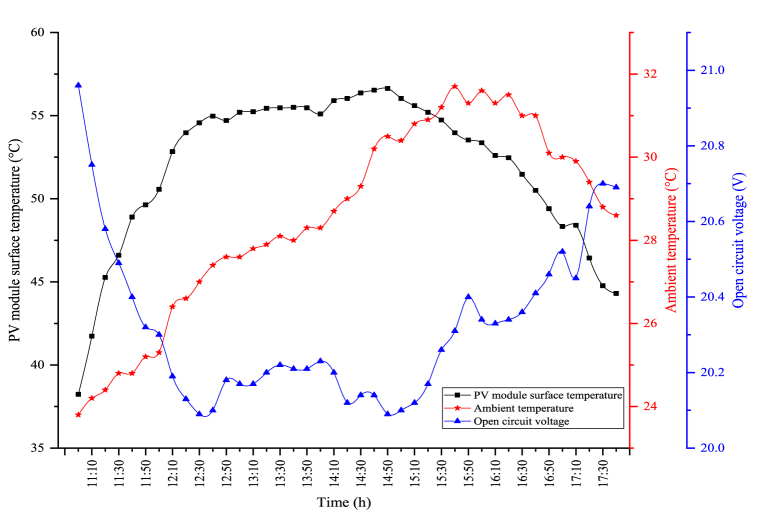
Changes in open circuit voltage and module temperature of the PV/T-VCRS (35 °C).

The changes between the open circuit voltage and the PV module's surface temperature obtained due to the experiment in the PV/T-VCRS (35 °C) are shown in Fig. 14. In the figure, there is an adverse correlation between the open circuit voltage and the module temperature. The maximum PV module temperature in the PV/T-VCRS (35 °C) is determined as 56.63 °C. During the experiment period, the average module temperature is calculated as 52 °C. The maximum open circuit voltage was determined to be 20.96 V according to the low module temperature. During the experiment period, the average open circuit voltage was determined as 20.32 V.

**Fig. 15:**
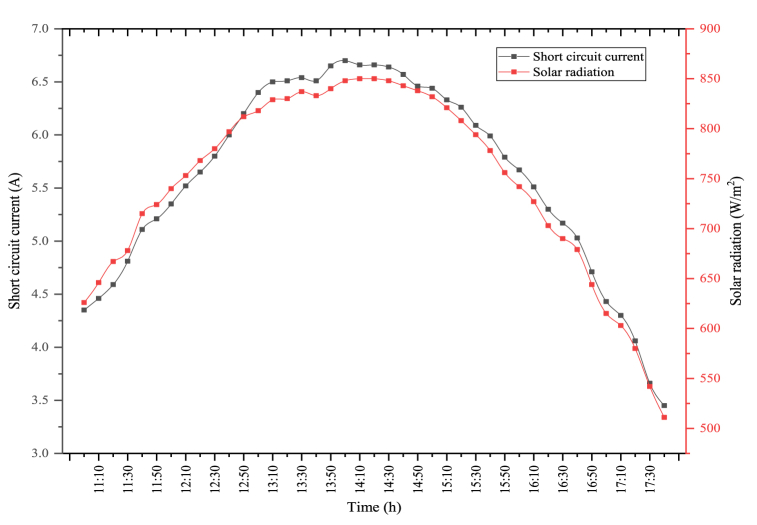
Changes in short circuit current and solar radiation of the PV/T-VCRS (35 °C).

The changes between short circuit current and solar radiation because of the experiment performed in the PV/T-VCRS (35 °C) are shown in Fig. 15. The change between the two parameters acts similarly to each other. Maximum solar radiation and average solar radiation are observed as 850 W/m^2^ and 746.22 W/m^2^ in the PV/T-VCRS (35 °C). Solar radiation is important in terms of the module's power and electrical efficiency. Because the amount of solar radiation coming to the module surface directly affects the amount of short circuit current generated in the module. The maximum short circuit current and the average short circuit currents are determined respectively to be 6.66 A and 5.61 A in the PV/T-VCRS (35 °C).

**Fig. 16:**
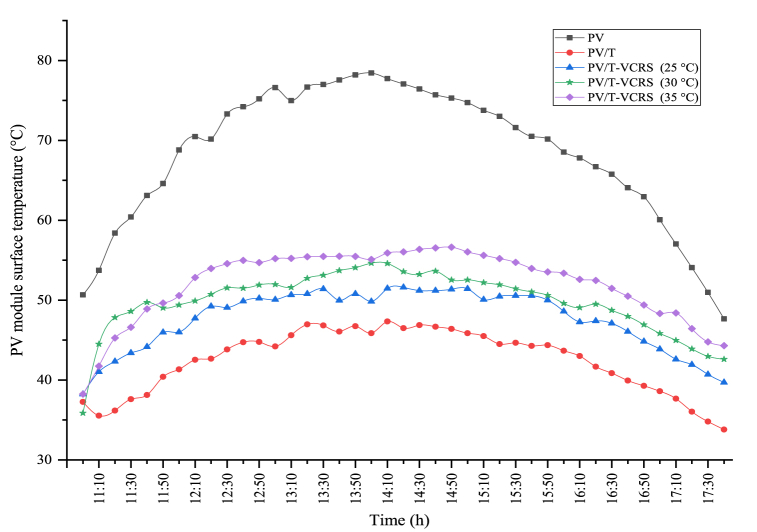
Changes in the PV module's surface temperature in different circumstances.

The module temperature directly affects the open circuit voltage produced in the PV module. In this circumstance, it directly influences the electrical efficiency and power of the PV module. The module temperature in the experiments carried out in different circumstances is given in Fig. 16. In the figure, the maximum module temperature for the PV, the PV/T, and the PV/T-VCRS (25 °C, 30 °C, and 35 °C) respectively 78.43 °C, 47.33 °C, 51.6 °C, 54.63 °C, and 56.63 °C were obtained. During the experiment period, the average temperature for the PV module was 68.39 °C, the average temperature for the PV/T module was 42.43 °C, the average temperature of the PV/T-VCRS at 25 °C was recorded to be 47.58 °C, the average temperature the PV/T-VCRS at 30 °C is recorded to be 49.8 °C, and the average temperature the PV/T-VCRS at 35 °C is 51.99 °C.

**Fig. 17:**
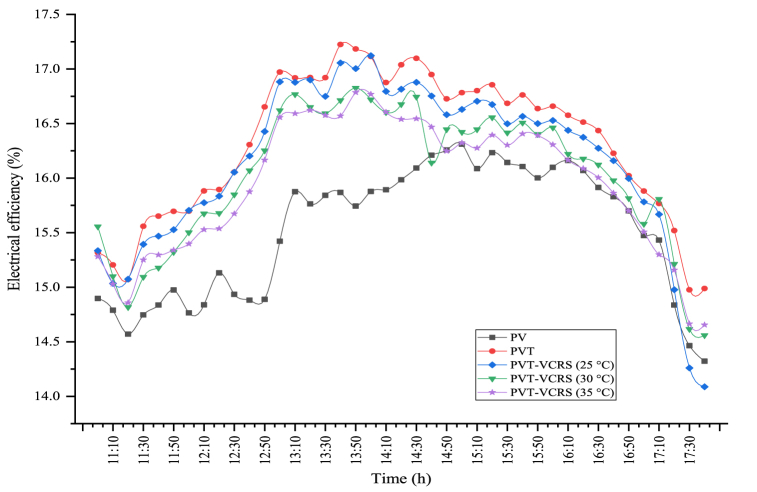
Changes in electrical efficiency in different circumstances.

Electrical efficiency is the most important assessment parameter of the PV module's performance. The electrical efficiencies of the PV, the PV/T, and the PV/T-VCRS at 25 °C, 30 °C, and 35 °C are shown in Fig. 17. The maximum electrical efficiencies were obtained respectively for the PV, the PV/T, and the PV/T-VCRS at 25 °C, 30 °C, and 35 °C as 16.31 %, 17.22 %, 17.12 %, 16.82 %, and 16.79 %. Average electrical efficiency has been determined for the PV, the PV/T, and the PV/T-VCRS at 25 °C, 30 °C, and 35 °C 15.52 %, 16.32 %, 16.15 %, 16.05 %, and 15.94 %, respectively. The significant reason for the higher electrical efficiency in PV/T-VCRS compared to the module is that the surface temperature of the module is lower because of the cooling water flowing behind it. The electrical efficiency of the module is defined as this causes the power of the module to enhance throughout the experiment.

**Fig. 18:**
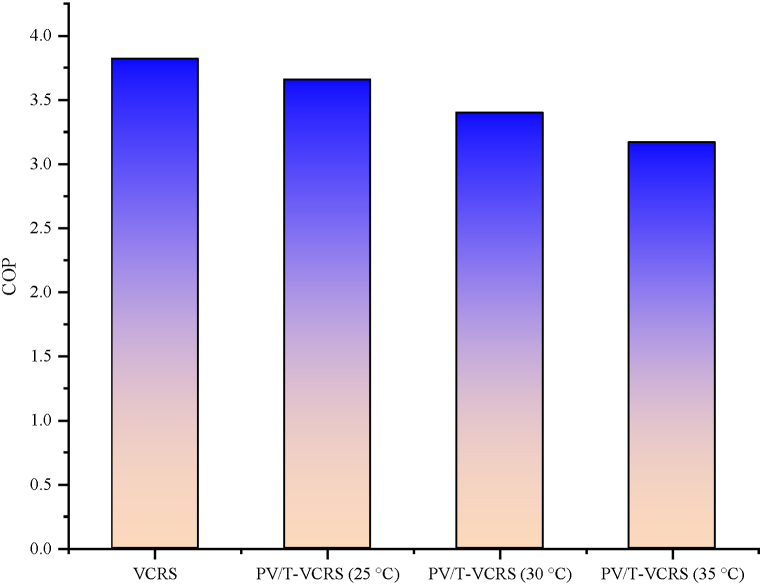
Changes in COP value of the VCRS and the PV/T-VCRS at different temperatures.

The most significant parameter to evaluate the VCRS's performance is the COP. Indicating the enhancement in COP is the quantity of heat removed from the condenser. When more heat is transferred from the condenser, the faster the refrigeration procedure goes on, the less power the compressor consumes. The quantity of heat discharged from the condenser decreases, as the water tank temperature increases. This is because the temperature variation between the refrigerant and the cooling water reduces due to the enhancement in temperature of the cooling water coming to the condenser. The quantity of heat distributed by the condenser is further reduced due to the decrease in the temperature difference. This is one of the reasons why COP values in combined refrigeration systems are lower than VCRS. Another reason is that as the heat released amount from the condenser decreases, a compressor is forced more to cool. In this circumstance, it increases the system's energy consumption. The enhancement in energy consumption directly affects the reduction in the COP. In PV/T-VCRS, as the water tank temperature increases, the heat and energy consumption from the condenser increases. For this reason, a lower COP value was obtained in combined systems [58]. The COP values is given in Fig. 18. The COP values of VCRS and PV/T-VCRS at 25 °C, 30 °C, and 35 °C were determined as 3.82, 3.66, 3.4, and 3.17, respectively.

**Fig. 19:**
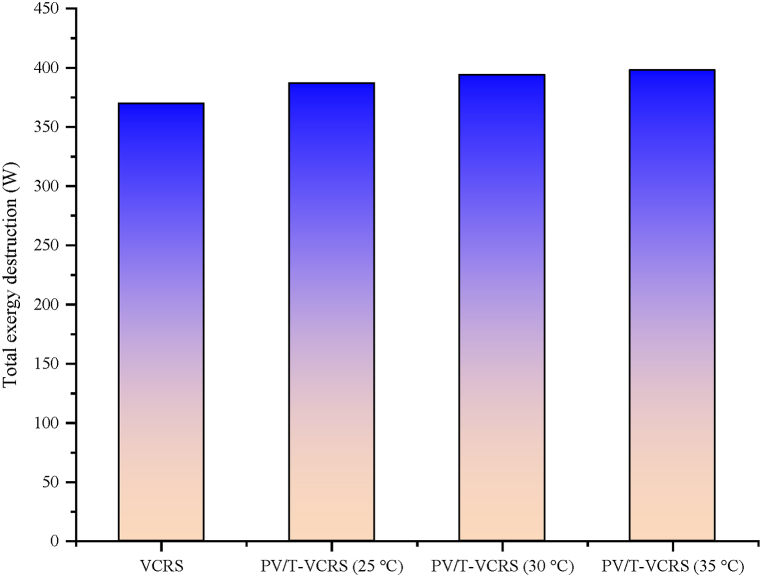
Changes in total exergy destruction of VCRS and PV/T-VCRS at 25 °C, 30 °C, and 35 °C.

As indicated in Fig. 19, the exergy destruction is for VCRS and PV/T-VCRS at 25 °C, 30 °C, and 35 °C as 370 W, 387 W, 394 W, and 398 W, respectively. Exergy destruction was less since the quantity of heat dissipated from the condenser in PV/T-VCRS is higher than in VCRS. The reason for this is that the flow rate of the water passing around the condenser enhances when the compressor outlet temperature enhances over the set temperature (40 °C) in VCRS. Thus, the quantity of heat to be discharged by the condenser is enhanced. Hence, exergy destruction's amount in VCRS is also less.

**Fig. 20:**
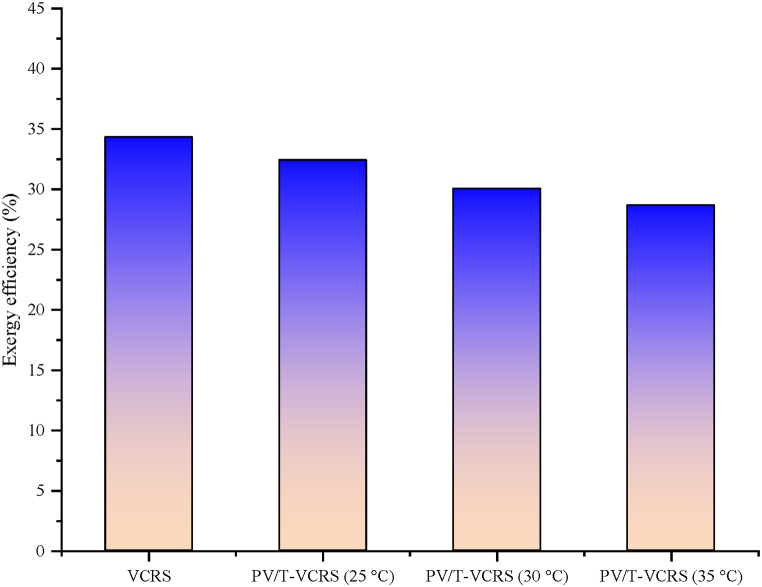
VCRS and PV/T-VCRS at 25 °C, 30 °C, and 35 °C exergy efficiency changes.

Exergy efficiencies of VCRS and the PV/T-VCRS at 25 °C, 30 °C, and 35 °C are given in Fig. 20. The exergy efficiency was calculated as 34.33 % in VCRS and 32.42 %, 30.06 %, and 28.67 % in PV/T-VCRS (25 °C, 30 °C, and 35 °C), respectively. The reason for achieving high exergy in VCRS is that total exergy destruction is lower. This circumstance indicates that VCRS operates more efficiently than PV/T-VCRS. A higher amount of heat released from the condenser affects the COP value positively. This value directly affects the total exergy loss and exergy efficiency. The water temperature used during the experiment in VCRS is approximately 20 °C. However, since the set temperatures are 25 °C, 30 °C, and 35 °C while performing experiments in PV/T-VCRS, less heat transfer takes place between the fluids as the temperature diversity between the water coming from the water tank temperature and the condenser temperature decreases. The condenser releases less heat due to this circumstance. Less heat dissipation of the VCRS causes the compressor to consume more energy.

**Fig. 21:**
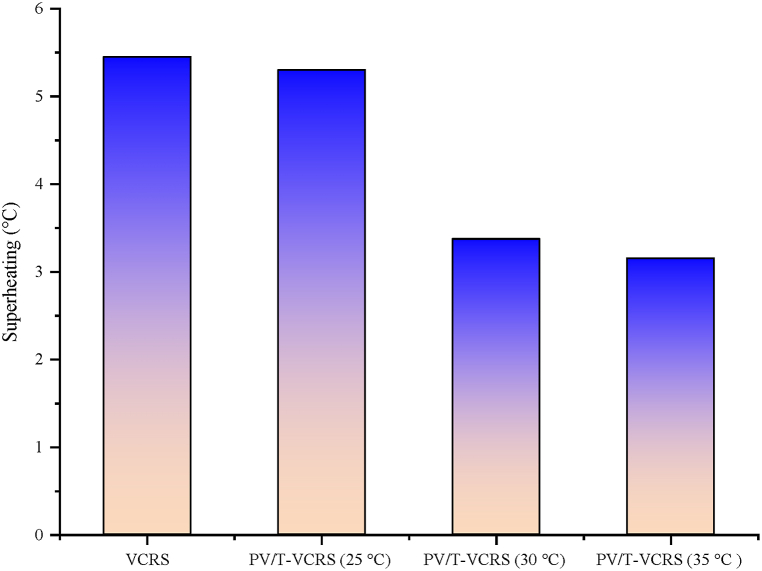
Changes in superheating of VCRS and PV/T-VCRS at 25 °C, 30 °C, and 35 °C.

Superheating is the method commonly used in VCRs. In the superheating process, which is widely used in conventional methods, the refrigerant is heated a little more with a heat exchanger placed in the evaporator outlet and the condenser outlet. The purpose of applying this process is to prevent the refrigerant entering the compressor from entering in liquid form. If this circumstance is not prevented, the liquid refrigerant will cause serious damage to the compressor and the compressor life will be reduced. In this study, a new method was tried in contrast to the conventional superheating method. This method is PV/T module-assisted superheating. Heat transfer takes place because of the temperature diversity between the water coming from the PV/T module and the refrigerant coming from the evaporator by using a heat exchanger for superheating. Superheating values obtained in VCRS and PV/T/VCRS are indicated in Fig. 21. The superheating in VCRS are obtained at 5.45 °C, PV/T-VCRS (25 °C, 30 °C, and 35 °C) at 5.3 °C, 3.37 °C, and 3.15 °C, respectively. It is seen that the superheating process reduces with the increment of the water tank's set temperature. However, it was observed that the values in the conventional method were also obtained with the method used in this study.

**Fig. 22:**
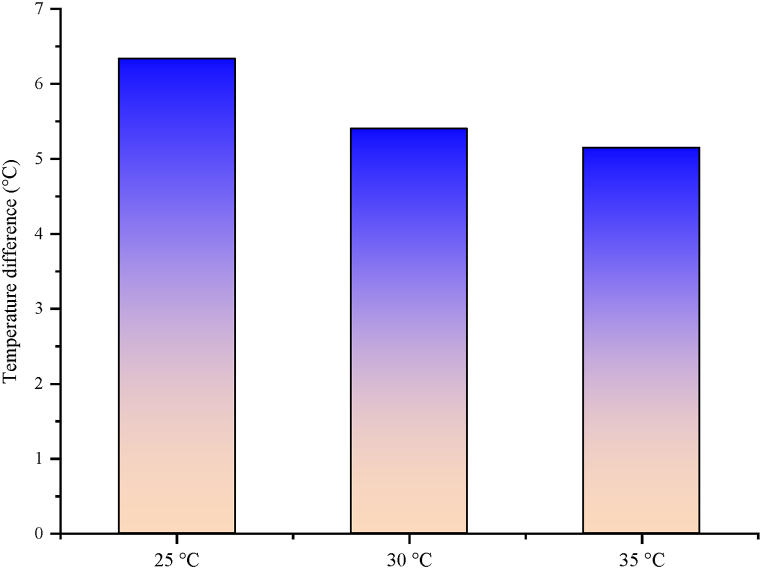
Coolant inlet and outlet temperature differences in the internal heat exchanger in PV/T-VCRS.

The importance of superheating in VCRS was explained in previous sections. It will be even more helpful to see the inlet and outlet water temperatures of the water coming from the PV/T module to the internal heat exchanger to fully examine the circumstances in the superheating process. The outlet and inlet water temperature difference to the internal heat exchanger is indicated in Fig. 22. As shown in the figure, the trend moves in parallel with the superheating process. The cooling water outlet and inlet temperature diversity in PV/T-VCRS at 25 °C, 30 °C, and 35 °C were obtained as 6.338 °C, 5.4, and 5.15, respectively. In other words, there has been heat transfer from the cooling water to the refrigerant in the internal heat exchanger. Because of the heat exchanger, the cooling water's temperature decreases. It is seen that the diversity in this input and output reduces with the enhancement of the water tank's set temperature. This circumstance directly explains the reason why the superheating process temperature reduces as the temperature enhances in the superheating process. The temperature diversity between the cooling water and refrigerant decreases as the set temperature increases. This reduces the heat transfer between the two fluids. In this circumstance, it affects the cooling water outlet and inlet temperature difference.

Having a good performance of a system is not the only evaluation criterion of that system. The PV/T-VCRS's economic performance is as important as its performance. PV/T module performances, thermoeconomic, and enviro-economic parameters of the PV/T-VCRS at different temperatures are shown in Table 3. The PV/T module's thermal efficiency in the PV/T-VCRS at 25 °C, 30 °C, and 35 °C was determined as 40.8 %, 38.4 %, and 35.9 %, respectively. Nevertheless, the PV/T module's overall efficiency was obtained as 56.95 %, 54.45 %, and 51.84 %, respectively. The PV/T module's exergy destruction in the PV/T-VCRS at 25 °C, 30 °C, and 35 °C was determined as 339.39 W, 348.37 W, and 375.24 W, respectively. However, the PV/T module's exergy efficiency was obtained as 37.7 %, 35.15 %, and 32.34 %. The energy cost of the PV/T-VCRS at 25 °C, 30 °C, and 35 °C was obtained as 0.359 $/kWh, 0.443 $/kWh, and 0.508 $/kWh, respectively. Therefore, because of the thermoeconomic analysis, the $R_{ex}$ value was determined as 0.647 kWh/$, 0.686 kWh/$, and 0.692 kWh/$ in the PV/T-VCRS at different temperatures, respectively. Because of enviro-economic analysis, 15.17 ¢/h, 16.52 ¢/h, and 17.6 ¢/h were obtained for the PV/T-VCRS at 25 °C, 30 °C, and 35 °C.

**Table 3:** PV/T efficiency and economic parameters in PV/T-VCRS.

Experiment circumstances	$\eta_{el}$ (%)	$\eta_{th}$ (%)	${\dot{Ex}}_{dest.}$ (W)	$\eta_{ex}$ (%)	$Z_{{CO}_{2}}$ (¢/h)	Energy Cost ($/kWh)	$R_{ex}$ (kWh/$)
PV/T-VCRS (25 °C)	16.15	40.8	339.39	37.7	15.47	0.359	0.647
PV/T-VCRS (30 °C)	16.05	38.4	348.37	35.15	16.52	0.443	0.686
PV/T-VCRS (35 °C)	15.94	35.9	375.24	32.34	17.6	0.508	0.692

**Fig. 23:**
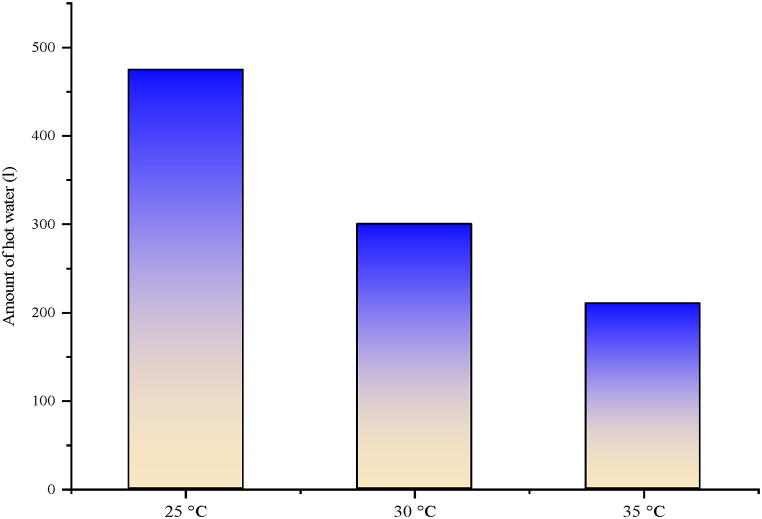
The amount of hot water obtained in PV/T-VCRS.

One of the advantages of PV/T-VCRS's performance is the availability of hot water. The set temperature in the water tank was set to 25 °C, 30 °C, and 35 °C and hot water was obtained at these temperatures in PV/T-VCRS. The quantity of water obtained from different water tank set temperatures is indicated in Fig. 23. In the figure, as the set temperature value enhances, the amount of hot water obtained decreases. 475 L domestic hot water at set temperature 25 °C, 300 L at 30 °C, and 210 L at 35 °C were obtained.

## 4 Conclusion

Today, energy is of major importance. The need for energy is enhancing day by day with the enhancement in technology and population. However, most of the energy needed is supplied by fossil fuels. Scientists started to investigate more environmentally friendly alternative energy sources, due to the enhancement in the demand for fossil fuels, their negative impacts on the environment, and the reduction in their reserves. Because of these studies, the increasing interest in renewable energy sources as alternative energy sources to fossil fuels is increasing day by day. In recent years, studies on solar energy have attracted attention. Studies on solar energy have not only been used alone but have started to be carried out by integrating them into different thermal systems. In this study, the solar energy and cooling system were not used separately as in previous studies but were used as a hybrid. The focus was on increasing the performance of both systems by operating them together. The findings from the hybrid system are as follows.

❖ The PV module's surface temperature reaches high temperatures, negatively affecting the module's electrical efficiency. Consequently, it is tried to hold the module temperature at low temperatures or constant. In this study, water is passed behind the module to keep the module temperature at low levels. There was a decrease of 30.43 %, 27.18 %, and 23.98 % of the module temperature in PV/T-VCRS at different temperatures (25 °C, 30 °C, and 35 °C) compared to the PV module, respectively.
❖ Keeping the module temperature at low temperatures increases the PV module's electrical efficiency. An enhancement in electrical efficiency is detected in PV/T-VCRS (25 °C, 30 °C, and 35 °C) compared to the PV module, 4.06 %, 3.41 %, and 2.71 %, respectively.
❖ Total exergy destruction is determined to be 387 W, 394 W, and 398 W for the refrigeration system in PV/T-VCRS at 25 °C, 30 °C, 35 °C, and 370 W in VCRS. It increased the system with lower exergy destruction in VCRS, while the system needed development in PV/T-VCRS at 25 °C, 30 °C, and 35 °C. Exergy efficiency was obtained to be 34.33 % in VCRS and 32.42 %, 30.06 %, and 28.67 % in PV/T-VCRS (25 °C, 30 °C, and 35 °C).
❖ Superheating is used to prevent VCRS from going to the compressor in liquid form. In this system, unlike conventional superheating methods, a PV/T module was utilized for superheating. Superheating with a PV/T module has a close performance compared to conventional superheating. The superheating temperature between the inlet and outlet temperature of the internal heat exchanger is respectively 5.45 °C, 5.3 °C, 3.37 °C, and 3 °C in VCRS and PV/T-VCRS at different temperatures.
❖ PV/T module's thermal efficiencies in PV/T-VCRS at 25 °C, 30 °C and 35 °C are 40.8 %, 38.4 %, and 35.9 %, respectively. However, the PV/T module's total efficiency was obtained as 56.95 %, 54.45 %, and 51.84 %. When the PV/T module was utilized alone, thermal and overall efficiencies were determined as 42.55 % and 58.87 %, respectively.
❖ The PV/T module's exergy destruction in PV/T-VCRS at 25 °C, 30 °C, and 35 °C was determined as 339.39 W, 348.37 W, and 375.24 W, respectively. However, the PV/T module's exergy efficiency was acquired as 37.7 %, 35.15 %, and 32.34 %. When the PV/T module was utilized alone, exergy destruction and exergy efficiency were determined as 315.92 W and 40.1 %, respectively.
❖ The energy cost of PV/T-VCRS at 25 °C, 30 °C, and 35 °C was obtained as 0.359 $/kWh, 0.443 $/kWh, and 0.508 $/kWh, respectively. Therefore, because of the thermoeconomic analysis, the $R_{ex}$ value was determined as 0.647 kWh/$, 0.686 kWh/$, and 0.692 kWh/$ in PV/T-VCRS at different temperatures, respectively.
❖ As a result of enviroeconomic analysis, 15.17 ¢/h, 16.52 ¢/h, and 17.6 ¢/h were obtained for PV/T-VCRS at 25 °C, 30 °C, and 35 °C.

In this study, it was seen that it gave better results than PV/T-VCRS when VCRS and PV/T modules were evaluated separately. Good performance could not be obtained in PV/T-VCRS since they are used separately in COP, PV/T module thermal efficiency, and electrical efficiency. However, close results were obtained. In particular, results very close to these performances were obtained when the set temperature was 25 °C in PV/T-VCRS. Besides, many benefits have been obtained from PV/T-VCRS. The first advantage is the superheating. The superheating was provided with the support of the PV/T module. In addition, hot water was acquired from the hybrid system at 25 °C, 30 °C, and 35 °C. Another advantage of this system is that the electricity needs of all components operating in the system during the experiment period, excluding the compressor, were supplied by the electricity produced from the PV/T-VCRS.

## Data and code availability statement

No data was used for the research described in the article.

## CRediT authorship contribution statement

**Gökhan Yıldız:** Writing – review & editing, Writing – original draft, Validation, Resources, Methodology, Investigation, Funding acquisition, Formal analysis, Data curation, Conceptualization. **Ali Etem Gürel:** Writing – review & editing, Writing – original draft, Supervision, Methodology, Investigation, Formal analysis, Data curation, Conceptualization. **Ferzan Katırcıoğlu:** Writing – review & editing, Writing – original draft, Software, Resources, Methodology, Funding acquisition, Data curation, Conceptualization. **Ümit Ağbulut:** Writing – review & editing, Writing – original draft, Methodology, Investigation, Conceptualization.

## Declaration of competing interest

The authors declare the following financial interests/personal relationships which may be considered as potential competing interests: The author ÜA serves as an AE in Heliyon.
